# Research on high-quality development path of strategic emerging enterprises enabled by innovation

**DOI:** 10.1371/journal.pone.0328918

**Published:** 2025-08-01

**Authors:** Lin Li, Wenjing Che, Qiangqiang Yao, Yujie Cui

**Affiliations:** 1 College of Economic and Management, Heilongjiang Bayi Agricultural University, Daqing, China; 2 Dongbei University of Finance and Economics, Dalian City, Liaoning Province, China; Huaqiao University, CHINA

## Abstract

Promoting the high-quality development of strategic emerging enterprises is an inevitable approach to building a strong nation, and innovation serves as the critical engine providing core momentum for such development. From the perspective of complex causal effect analysis, this article selects 176 A-share listed companies in strategic emerging industries from 2012 to 2023 as samples and employs a combination of methods including NCA, multi-period fsQCA, and empirical regression analysis to distill practical pathways for innovation-driven high-quality development in these enterprises. The research findings are summarized as follows: (1) There is no single necessary condition for achieving high-quality development in strategic emerging enterprises; rather, it is the result of the synergistic interaction among technological innovation, talent innovation, and policy innovation. (2) Technological innovation has consistently played a pivotal role across all periods, with R&D investment identified as a key factor driving high-quality development. Other contributing factors also exhibit heterogeneous effects within the configurations of each period. (3) Four distinct configuration paths for high-quality development emerge across the three periods: the technology-dominant type, the “technology and talent” dual-driven type, the “technology and policy” dual-driven type, and the comprehensive innovation type. This study leverages complex causal effect analysis to offer scientific insights to relevant policymakers and enterprise managers, thereby facilitating the high-quality development of strategic emerging enterprises.

## 1 Introduction

High-quality development, as the central development concept of socialism with Chinese characteristics in the new era, underscores sustaining a rational economic growth rate while placing greater emphasis on the efficiency, fairness, sustainability, and innovation of development. It serves not only as a new standard established by China for its future economic and social progress but also as the inevitable pathway to guiding the nation toward becoming a modernized power. This signifies that China’s economy has transitioned into a new phase, characterized by enhanced quality and efficiency. Pursuing high-quality development constitutes both a fundamental requirement and a critical objective for China’s economic advancement. The key to fostering high-quality development lies in constructing a modernized economic system. Given that industries are the backbone of the economy, the level of their modernization is directly correlated with the competitiveness and developmental potential of the overall economy. Strategic emerging enterprises, as core entities within strategic emerging industries, focus on these industries, which are underpinned by significant breakthroughs in cutting-edge technologies and substantial developmental demands, and play a pivotal leading and driving role in the comprehensive and long-term development of the economy and society.

For strategic emerging enterprises, addressing the rapid changes in current policies and the external market environment while promoting high-quality development has become a pressing issue. In particular, investments in technological innovation, talent development, and policy reform serve as critical strategies for these enterprises to adapt to technological and industrial transformations. Such investments not only facilitate the high-quality development of enterprises but also contribute to achieving sustainable economic growth. The report of the 19th National Congress of the Communist Party of China explicitly emphasized the necessity of high-quality development, signifying a transition from high-speed growth to a phase of high-quality development in China’s economy. This shift reflects a reorientation of the economic growth model from speed-centric to quality-focused. Studies have demonstrated that innovation significantly enhances the high-quality development of enterprises [1,2]. As the primary driver of high-quality development, innovation plays a pivotal role in shaping the trajectory of strategic emerging enterprises. Key questions remain regarding which innovative elements can effectively promote high-quality development, the nature of interaction effects among various innovative factors, and whether differences exist in the practical pathways through which innovation empowers high-quality development under diverse circumstances.

In the literature on high-quality development, a significant portion of research focuses on elucidating the fundamental essence and underlying logic, aiming to address the question of “What is high-quality development” [3]. Another group of scholars adopts a singular perspective to delve into the driving factors behind achieving high-quality development in strategic emerging industries [4]. However, there is a relative dearth of studies from the lens of complex system theory that explore the multidimensional logical mechanisms and practical strategies for attaining high-quality development in strategic emerging industries, i.e., “how to achieve it”. On one hand, domestic scholars primarily evaluate high-quality development based on novel developmental concepts. On the other hand, when investigating influencing factors, scholars consider digital economy [5,6] and technological innovation [7,8] as fundamental elements for promoting the high-quality development of enterprises. They conduct both quantitative and qualitative analyses using specific regional samples.

Upon reviewing existing literature, it is evident that most studies excessively focus on the “average net effect” of individual influencing factors on the high-quality development of strategic emerging enterprises while neglecting potential interactions and linkages among various factors. In reality, achieving high-quality development in strategic emerging industries is a complex, dynamic, and non-linear process wherein multiple factors intertwine to produce complementary or substitutive effects [9]. At present, some scholars have used QCA method to explore that the realization of high-quality development of enterprises needs the joint action of many factors [10]. Therefore, traditional regression methods cannot fully explain this complex causal configuration problem. In view of this, the paper uses 176 A-share listed companies of strategic emerging enterprises in 2020–2022 as panel data, Necessary Condition Analysis (NCA) and Qualitative Comparative Analysis (QCA) were combined for analysis and empirical regression are combined to analyze the complex causal relationship and linkage effect between innovation and high-quality development of strategic emerging enterprises, and provide referential solutions for the practical path of high-quality development of strategic emerging enterprises. This paper aims to answer the following questions: (1) Whether and to what extent are technological innovation, talent innovation and policy innovation the necessary conditions for high-quality development of strategic emerging enterprises? (2) What are the driving paths to achieve high-quality development of strategic emerging enterprises?

The marginal contribution of this study is as follows: First, based on the perspective of configuration, it analyzes the practical path of technology innovation, talent innovation and policy innovation to the high-quality development of strategic emerging enterprises, and provides new ideas for the empirical study of innovation empowerment; Second, the combination of NCA, fsQCA and empirical regression can alleviate the relatively single limitation of traditional econometric analysis to a certain extent; Third, the research conclusions will provide a referential practice path for national and local governments to promote the high-quality development of strategic emerging enterprises, which is also of great significance for China’s research on promoting high-quality development.

## 2 Literature review and theoretical analysis

### 2.1 Literature review

#### 2.1.1 Technological innovation and high-quality development.

Technological innovation refers to the creation of new technology or the utilization of scientific and technological knowledge and resources. It encompasses various aspects such as digital technology, research and development investment, and innovation output [11,12]. The integration of digital technology can reshape the enterprise’s innovation model by reducing innovation risks [13],increasing input in innovation activities, lowering financing costs, improving innovation output, and facilitating high-quality development [14,15]. Some scholars have proved that R&D investment is an important means for enterprises to obtain core technologies and realize technological innovation, and increasing R&D investment can improve enterprises’ independent innovation ability [16,17]. Additionally, some scholars propose that an enterprise’s technological innovation ability can be represented by its level of innovation output [18]. A higher level of innovation output indicates stronger technological innovative capacity and a greater degree of high-quality development for the enterprise. Some scholars also regard technological innovation as one of the main driving forces for economic growth [19]. Therefore, it is evident that technological innovation serves as a fundamental driving force for promoting high-quality development by efficiently leveraging disruptive technologies and cutting-edge advancements.

#### 2.1.2 Talent innovation and high-quality development.

High-quality development necessitates innovation as the primary driving force, with talent innovation serving as a crucial means to accomplish this objective. Talent innovation refers to the continuous discovery and cultivation of available talents within an organization through effective methodologies. Existing research primarily focuses on R&D personnel and highly educated individuals when it comes to talent innovation. R&D personnel serve as a fundamental basis for enterprises to enhance their innovative capabilities by proficiently identifying and analyzing problems, proposing innovative solutions from diverse perspectives, challenging traditions, and actively exploring new technologies and methods to inject fresh impetus into enterprise upgrading and technological innovation [20]. Highly educated individuals are distinguished by their profound professional knowledge and skills, possessing a solid theoretical foundation along with extensive practical experience. Through ongoing study and practice, they can continuously enhance their professional quality while providing substantial knowledge support for high-quality development. It is evident that the multidimensional characteristics of innovative talents contribute significantly to the core competitiveness of high-quality development while serving as the key cornerstone for enterprises in promoting such advancement.

#### 2.1.3 Policy innovation and high-quality development.

Innovation and development are crucial drivers for accelerating high-quality development, which necessitates robust support from national policies. The synergistic impact of government innovation subsidies and tax incentives can facilitate enterprise innovation and development. Government innovation subsidies have a positive incentivizing effect on enterprise innovation activities. On one hand, they can address the spillover effects of knowledge and technology in innovation activities by providing direct funds to compensate for any additional losses [21]. On the other hand, they can alleviate financial pressures faced by enterprises, share the risks associated with R&D failures, and reduce capital costs related to developing new technologies through financial support; thus relieving financing burdens on enterprises and promoting their innovative capabilities [22]. Some scholars have found that government subsidies provide additional financial support for enterprises, ease their financing constraints and enable them to invest more resources in innovation activities [23,24]. The implementation of preferential tax policies represents the most direct form of government support for promoting enterprise innovation as it reduces taxes paid by science and technology companies, enhances corporate profits, eases financial pressures on enterprises, and encourages continued investment in research and development [25]. It is evident that policy innovations can better meet China’s new requirements for scientific and technological advancement in this era while playing a significant role in stimulating enterprise innovation vitality and fostering high-quality development.

**Fig 1:**
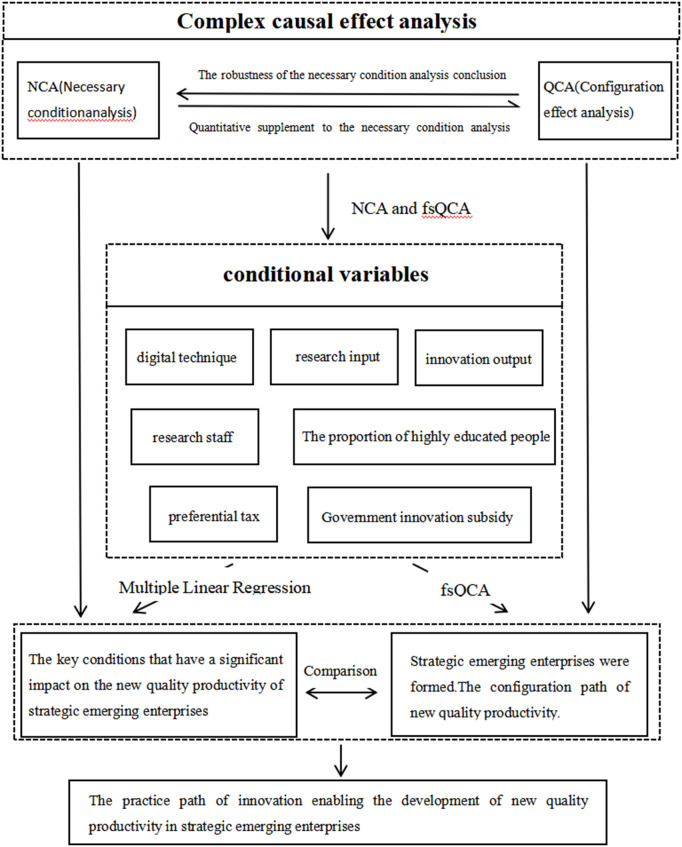
Configuration effect of innovation enabling high-quality development of strategic emerging enterprises.

The above theory indicates that under the impetus of policies, talent innovation and technological innovation interact and cooperate with each other, serving as crucial drivers for enterprises to achieve high-quality development. It is difficult to enhance the high-quality development of enterprises relying solely on a single factor. Policy support provides enterprises with a favorable external environment and resource guarantee, while talent innovation and technological innovation drive enterprises to keep making progress and breakthroughs internally. The three complement each other, forming an organic whole and jointly promoting the high-quality development of the enterprise. Therefore, from the perspective of configuration, the article further refines the three levels of policy innovation, talent innovation and technological innovation, and divides them into seven specific conditional variables to analyze the interaction and influence of each factor more comprehensively and meticulously. On this basis, this paper adopts the method combining NCA (Necessary Condition Analysis), fsQCA (Fuzzy Set Qualitative Comparative Analysis) and empirical regression to deeply explore the multiple driving paths and linkage effects of the high-quality development of strategic emerging enterprises. The specific research framework is shown in Fig 1.

### 2.2 Theoretical analysis

The essence of the complex causality theory resides in abandoning the simplistic and isolated modes of thinking prevalent in traditional causal analysis. Instead, it advocates treating the research object as a highly complex and dynamic system. Within this system, the various elements are not independent but rather intricately interconnected and mutually influential. Through extensive interactions and feedback mechanisms, these elements collectively construct intricate and diverse causal pathways. These pathways form an invisible yet sophisticated network, where even minor changes at any node can trigger a cascade of chain reactions, ultimately resulting in substantial differences in outcomes. Configuration theory similarly emphasizes adopting an integrative and systematic perspective. It posits that in real-world scenarios, a single factor rarely serves as a necessary condition for a specific outcome. More often, the outcome arises from the combination and synergistic effects of multiple factors configured in a particular manner. By juxtaposing configuration theory with complex systems theory, it becomes evident that both approaches “converge on the same destination through different routes.” Both theories acknowledge the complexity and systematic nature of the real world, stressing that factors should not be examined in isolation but rather their interrelationships and overall impacts should be prioritized. When analyzing strategic emerging enterprises through the lens of innovation, their high-quality development is not determined by a singular, isolated factor but is instead propelled by the concerted influence of multiple factors such as technological innovation, talent innovation, and policy innovation.

In conclusion, the deep integration of complex causality theory and configuration theory offers a robust framework for elucidating the intrinsic logic underlying innovation-driven high-quality development in strategic emerging enterprises. Specifically, complex causality theory examines the intricate network of multiple intertwined and non-linear causal relationships between innovation factors and the developmental outcomes of strategic emerging enterprises, uncovering the differentiated impact pathways shaped by dynamic interactions among various factors. Meanwhile, configuration theory emphasizes the collaborative interplay among diverse innovation condition variables, highlighting that elements such as technological innovation, talent innovation, and policy innovation do not independently drive development but instead produce synergistic effects through specific combination patterns and structural configurations. Together, these two theories complement and reinforce each other, collectively revealing the internal mechanisms through which innovation enables high-quality development in strategic emerging enterprises via the integration of multiple pathways and systemic element synergy.

## 3 Research methods

### 3.1 Method selection

#### 3.1.1 fsQCA is combined with NCA...

The article employs a combined method of fsQCA and NCA to investigate the complex causal mechanisms underlying how innovation drives the high-quality development of strategic emerging enterprises. Qualitative Comparative Analysis (QCA), introduced by Ragin in 1986, integrates both qualitative and quantitative attributes, enabling an in-depth exploration of causal relationships and combination mechanisms among multiple factors. This method not only identifies which combinations of factors lead to specific outcomes but also elucidates the differences among various factor combinations. The NCA method serves as a research tool for identifying and detecting necessary but potentially insufficient conditions within data, quantitatively analyzing whether and to what extent the antecedent variable constitutes a necessary condition for achieving the outcome. By examining the relationship between antecedent and outcome variables, NCA clarifies which conditions are indispensable for the occurrence of the result, though they may not be sufficient on their own. This approach aids in pinpointing conditions that must be fulfilled under all circumstances, thereby strengthening the research foundation. Combining NCA with fsQCA enhances the scientific rigor of the research findings. Consequently, the article first evaluates whether innovation is a necessary condition for achieving high – quality development using the NCA method, followed by employing fsQCA to validate the robustness of this necessary condition analysis. Subsequently, from a configurational perspective, fsQCA is utilized to explore the influence pathways of different condition combinations on high-quality development outcomes, revealing how innovation impacts high-quality development through intricate causal mechanisms. Lastly, considering the dynamic nature of antecedent conditions, the temporal dimension is integrated into the QCA analysis.

#### 3.1.2 Empirical regression combined with NCA.

This paper first uses the causal model of traditional empirical regression $Y=a+b_{1}X_{1}+b_{2}X_{2}+b_{3}X_{3}+\cdots$ to verify the correlation between variables and high-quality development. In the NCA perspective, this necessary causal relationship should be reflected in the “multiplicative” model $Y=f\left(X_{1}\times X_{2}\times X_{3},\cdots \right)$. In this model, it is assumed that the absence of a certain necessary condition factor (i.e., when $X_{i}=0$) will result in a missing result (i.e., Y=0). This expression can effectively reveal the necessary causal relationship between the combination of conditions and the outcome from the perspective of complex causal effects.

### 3.2 Sample and data

The sample enterprises were mainly selected in accordance with the definition in the “Classification of Strategic Emerging Industries (2018)” and based on the enterprise qualification certification data in the CSMAR database. Other relevant data mainly come from the WIND database, annual reports of listed companies, and the National Intellectual Property Administration. The A-share listed companies of China’s strategic emerging enterprises from 2012 to 2023 were screened. After excluding the samples of ST, *ST and those delisted during the period, only the samples without data missing for 12 consecutive years were retained. Eventually, valid data of 176 listed companies were obtained, distributed across 26 provinces in China. The specific indicator system is shown in Table 1.

**Table 1:** Measurement and description of high-quality development variables of strategic emerging enterprises.

Variables	First-level indicators	Indicator measurement method description
Technological innovation	Digital technology	Digital transformation word frequency
	Research and development investment	LN(R&D investment)
	Innovation output	LN(Number of corporate patent applications +1)
Talent innovation	Research and development personnel	Research and development personnel account for the total number of employees
	The proportion of highly educated personnel	Personnel with bachelor degree or above/total personnel of the company
Policy innovation	Tax incentives	Various taxes paid/ (Various taxes paid + tax refunds received)*100
	Government innovation subsidies	LN (Government innovation subsidy for companies +1)
High-quality development		Total factor productivity

### 3.3 Measurement and calibration

#### 3.3.1 Result variable.

High-quality development. High-quality development entails a complex system wherein scientific and technological innovation drives the enhancement of total factor productivity, which essentially represents the efficiency of resource allocation and reflects an enterprise’s capacity to achieve maximum output given specific inputs. As a substitute variable for enterprises’ high-quality development, total factor productivity is measured in this study using the LP method (index estimation), building upon previous research [2].

#### 3.3.2 Conditional variables.

Digital technology. Learn from the practice of Bi M. et al. to extract the total vocabulary of the annual report [26]. Most existing literature measure digital transformation by the frequency of digital technology feature words in annual reports, reflecting the relative importance of listed companies to digitalization.

Research and development investment. R&D investment is the embodiment of enterprises’ innovation willingness and the prerequisite for improving enterprises’ innovation ability. According to the existing research, this paper uses the natural logarithm of enterprises’ R&D expenditure to measure R&D funds [27].

Innovation output. The number of patent applications is the direct result of innovation output of an enterprise, which can reflect its technological innovation ability [28,29]. Patent applications are divided into three types: invention, utility model and design. This paper uses the number of patents applied by an enterprise in the current year (the sum of the number of invention patents, utility model patents and design patents) to measure the innovation output of an enterprise in a certain period [30–32].

Research and development personnel. The number of R&D personnel can directly show the degree of support for new technology research and development of enterprises, which greatly affects the ability and quality of innovation of enterprises. This article uses the ratio of the number of R&D personnel to the total number of employees to measure the proportion of R&D personnel.

The proportion of highly educated personnel. The level of human capital of an enterprise can reflect the ability of an enterprise to absorb knowledge. This paper takes the ratio of the number of employees with a bachelor’s degree or above to the number of employees in an enterprise as the value of this index.

Tax benefits. Preferential tax policies have a significant impact on enterprises R&D investment and technological innovation [25]. The index of tax incentives in this paper is “all tax rebates received/ (all tax rebates received + all taxes paid)”.

Government innovation subsidies. Government subsidies refer to policy funds provided free of charge by the government to enterprises in order to achieve specific social and economic goals [33,34].This paper measures government innovation subsidies by adding logarithm to the actual amount of government innovation subsidies obtained by strategic emerging enterprises in this fiscal year [35], so as to reflect the government’s support for the development of new quality productive forces of strategic emerging enterprises.

#### 3.3.3 Calibration.

Table 2 shows the calibration anchor points of each variable. This paper calibrates the data with reference to the studies of Fiss and Fan [36–38], and sets the calibration standards of the three anchor points of full membership, cross and non-membership of each variable as 0.95, 0.5 and 0.05 point of descriptive statistics respectively. The calibrated data will be between [0,1], where 0 means no affiliation at all and 1 means full affiliation. In order to avoid unidentifiable situations, the calibrated data will be adjusted by adding 0.001 to the membership score of 0.5 [39].

**Table 2:** Calibration anchor information of each variable2.

Time	Time1			Time2			Time3		
Variables	Anchor Point								
	HP	CP	CUP	HP	CP	CUP	HP	CP	CUP
HQD	9.27	8.58	7.93	10.33	8.59	7.16	10.54	10.54	10.54
DT	88.99	2.63	0.00	191.25	9.00	0.00	8.66	8.66	8.66
RDI	20.15	17.98	16.62	21.17	19.08	17.20	7.14	7.14	7.14
IO	292.66	38.88	3.75	790.75	83.00	7.75	818.75	93.00	7.75
RDP	608.33	92.06	15.36	52.00	15.97	5.61	50.79	17.56	6.87
PHEP	0.78	0.28	0.08	0.79	0.36	0.09	0.84	0.42	0.11
TB	0.58	0.15	0.00	0.63	0.16	0.00	0.74	0.22	0.01
GIS	16.67	14.51	12.26	17.87	15.43	12.03	18.66	15.02	10.87

**Note1:** HP, CP and CUP are respectively Full membership points, Crossing points and Completely unaffiliated points;

**Note2:** HQD, DT, RDI, IO, RDP, PHEP, TB and GIS are respectively High quality development, Digital technology, Research and development investment, Innovation output, Research and development personnel, Proportion of highly educated personnel, Tax benefits and Government innovation subsidies,the same below.

## 4 Empirical analysis

### 4.1 Analysis of necessary conditions

**Fig 2:**
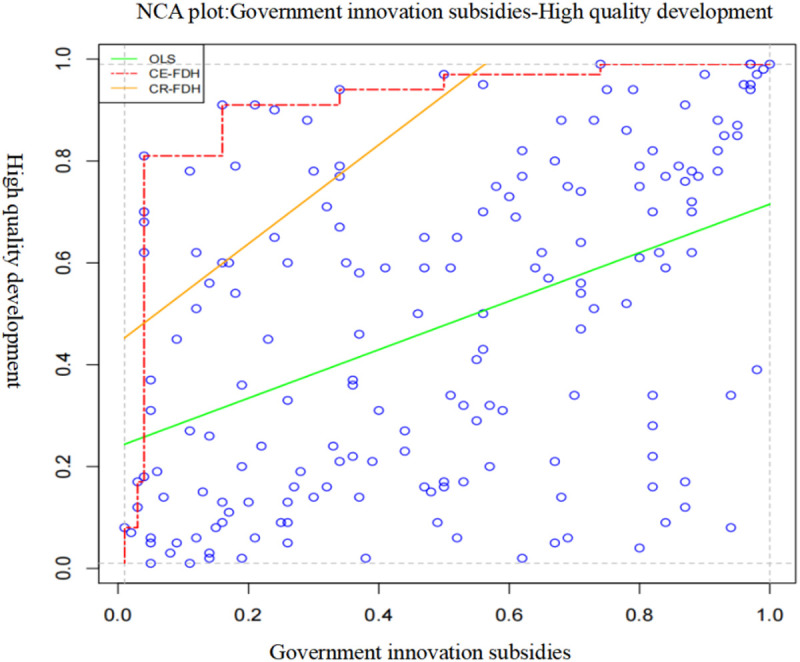
Analysis results of NCA single necessary conditions for Government innovation subsidies.

NCA includes upper limit regression analysis (CR) and upper limit envelopment analysis (CE) two estimation methods of upper limit range. The data used in this paper are continuous. The CR method is mainly used, and the results of the CE method are used for comparison. NCA method to determine the necessary conditions to meet the following conditions: first, the effect size d > 0.1; Second, the effect size test results of Monte Carlo simulation replacement are significant, that is, P < 0.05, the effect size is between 0 and 1, the larger the effect size is, the smaller the effect size is, the less than 0.1, the effect size is too small and there is no necessity [40]. Fig 2 indicates that government innovation subsidies can effectively influence the high-quality development of strategic emerging enterprises.

As can be seen from Table 3, the effect size of government innovation subsidies is greater than 0.1 and the test results are significant, but its accuracy is less than 95%, which cannot be identified as a necessary condition. The P-values of the other variables are all greater than the standard value 0.05, and the effect size d is less than 0.1, which does not meet the conditions of NCA necessity analysis. In summary, all the seven conditional variables studied in this paper are not necessary conditions for the high-quality development of strategic emerging enterprises.

**Table 3:** NCA necessary condition results analysis.

Conditional variables	Methods	Precision	Upper limit area	Range	Effect Size	P value
DT	CR	99.4%	0.008	0.940	0.008	0.148
	CE	100.0%	0.007	0.940	0.008	0.144
RDI	CR	100.0%	0.016	0.980	0.016	0.501
	CE	100.0%	0.032	0.980	0.033	0.087
IO	CR	94.3%	0.044	0.950	0.047	0.054
	CE	100.0%	0.043	0.950	0.045	0.000
RDP	CR	100.0%	0.000	0.970	0.000	0.957
	CE	100.0%	0.002	0.970	0.002	0.956
PHEP	CR	99.4%	0.009	0.940	0.010	0.631
	CE	100.0%	0.008	0.940	0.009	0.742
TB	CR	98.9%	0.017	0.940	0.019	0.297
	CE	100.0%	0.015	0.940	0.016	0.205
GIS	CR	90.9%	0.149	0.970	0.153	0.000
	CE	100.0%	0.075	0.970	0.078	0.000

**Note:** 0 < d < 0.1 means “low effect”; 0.1 < d < 0.3 means “medium effect”; d > 0.3 for “high effect”. The p-value represents the permutation test in the NCA, and the closer the p-value is to 0, the more significant the effect on the result.

The bottleneck analysis results of the NCA method are presented in Table 4, which further examines the conditional requirements necessary to achieve a specific level of outcomes. The findings indicate that to attain a high-quality development level of 50%, a minimum government innovation subsidy level of 6.2% is required, while no bottlenecks exist for other conditions. To achieve a 100% high-quality development level, tax incentives must be at least 19.7%, government innovation subsidies should reach 97.6%, innovation personnel reserve needs to be maintained at 87.1%, and research and development investment should be set at a minimum of 95.2%. No bottlenecks are observed for other conditions. These results demonstrate that individual conditional variables have limited impact on enterprise high-quality development, whereas the combined effect of multiple variables can lead to such development.

**Table 4:** Bottleneck level results analysis.

HQD	DT	RDI	IO	RDP	PHEP	TB	GIS
0	NN	NN	NN	NN	NN	NN	NN
10	NN	NN	NN	NN	NN	NN	NN
20	NN	NN	NN	NN	NN	NN	NN
30	NN	NN	NN	NN	NN	NN	NN
40	NN	NN	1.7	NN	NN	NN	NN
50	NN	NN	3.7	NN	NN	NN	4.9
60	NN	NN	5.7	NN	NN	NN	15.1
70	NN	NN	7.6	NN	NN	NN	25.3
80	NN	3.6	9.6	NN	NN	0.8	35.5
90	3.6	7.3	11.6	NN	2.1	9.3	45.7
100	10.9	11	13.6	2	17.3	17.8	55.8

**Note:** The table uses a cap regression analysis CR; NN indicates not necessary.NA = is no longer a necessary determinant.

### 4.2 Configuration analysis

The results of individual condition necessity analysis for the high-quality development of enterprises are presented in Table 5. In this study, fsQCA4.1 is employed to examine whether a single conditional variable serves as a necessary condition for the outcome variable. This approach also allows for testing the analysis findings of NCA. Notably, consistency serves as a crucial criterion for assessing necessity, while coverage determines the reliability of each condition variable when interpreting the outcome variable [41,42]. If the consistency value of a single condition exceeds 0.9, it indicates that the condition variable is necessary for achieving the outcome [43]. Conversely, if the consistency value falls below 0.9, it suggests that the condition variable is not essential for attaining the outcome [44]. As depicted in Figure X (or Table X), all conditions exhibit consistencies lower than 0.9, which aligns with NCA results and implies weak explanatory power of tax incentives, government innovation subsidies, innovation personnel reserve, proportion of highly educated talents, R&D investment and technological innovation on high-quality development in strategic emerging enterprises; thus indicating an absence of any singular necessary condition.

**Table 5:** Necessity analysis of individual conditions.

Condition variables	High quality development		Non-high-quality development	
	Consistency	Coverage	Consistency	Coverage
DT	0.589975	0.651188	0.55425	0.6825
~ DT	0.712345	0.58889	0.716733	0.661036
RDI	0.846737	0.849494	0.477211	0.534129
~ RDI	0.535641	0.478728	0.865532	0.863021
IOt	0.733382	0.83052	0.442517	0.55908
~IO	0.61065	0.495417	0.865855	0.783694
RDPl	0.626758	0.62548	0.587329	0.65391
~ RDP	0.653204	0.586572	0.663614	0.664832
PHEP	0.766318	0.737933	0.546062	0.586642
~ PHEP	0.570742	0.529851	0.756061	0.78306
TB	0.620387	0.646337	0.588837	0.684408
~ TB	0.697079	0.60312	0.695722	0.671555
GIS	0.603318	0.591933	0.695722	0.671555
~ GIS	0.69167	0.630852	0.627195	0.638197

**Note:** “~” indicates that the logical operation is not set, indicating that the variable is at a relatively low level.

The results of the combined analysis of all condition variables are shown in Table 6. With reference to Du Yunzhou, the case frequency value is set to 1.5% of the original case number, and the case number of the paper is 176, so the case threshold is set to 2; The original consistency threshold is set to 0.8 and the PRI consistency threshold is set to 0.8; In terms of the choice of analysis scheme, the paper uses fsQCA4.1 to obtain three results: reduced solution, intermediate solution and complex solution. Referring to existing literature, the paper chooses the intermediate solution as the main way and the reduced solution as the auxiliary way [45]. The conditions of the intermediate solution can be divided into core conditions and edge conditions. The core conditions exist in the intermediate solution and the reduced solution, and the edge conditions only exist in the intermediate solution.

**Table 6:** Results of configuration analysis for high-quality development of strategic emerging enterprises.

Variables	Time1		Time2						Time3						
	P1		P2			P3			P4		P5	P6			P7
	H1a	H2b	H2a	H2b	H2c	H3a	H3b	H3c	H4a	H4b	H5	H6a	H6b	H6c	H7
DT	⨂	⨂			⨂			⨂	⨂	⨂		●	●	●	
RDI	●	●	●	●	●	●	●	●	●	●	●	●	●	●	●
IO	●	●	●	●	●	●	●	●	●	●	●	●	●	●	●
RDP		●	●	●	●	⨂			⨂			⨂		●	●
PHEP	●		●	●	●	●	●	●		⨂		●	●	●	●
TB		⨂	⨂			●	●	●		⨂	●	⨂	⨂		●
GIS	●	●		⨂	⨂		⨂		⨂	⨂	●		⨂	⨂	
CS	0.94	0.95	0.96	0.94	0.97	0.96	0.95	0.95	0.96	0.96	0.96	0.97	0.98	0.98	0.98
RC	0.29	0.43	0.34	0.35	0.33	0.35	0.37	0.39	0.34	0.39	0.30	0.26	0.24	0.25	0.24
UC	0.04	0.17	0.02	0.01	0.00	0.00	0.00	0.00	0.02	0.06	0.01	0.01	0.01	0.00	0.00
OU	0.95		0.94						0.96						
OC	0.47		0.52						0.56						

**Note:** ● indicates the presence of a core condition, ● indicates the presence of an edge condition, ⨂ indicates the absence of a core condition, ⨂ indicates the absence of an edge condition, and a blank indicates the presence or absence of a condition.

The data in the table indicates that among the three periods, there are seven paths that can achieve a high-quality development level for strategic emerging enterprises. The consistency of each combination is close to 1, indicating that this conditional combination is significant and has a high explanatory power for the outcome variable. The coverage rate of the combined solution is about 0.5, indicating that the 7 paths cover about 50% of all the results, reaching a relatively high level and having certain research value. In order to present the results of fsQCA analysis more clearly and reasonably, based on the theoretical framework, this article further transforms the diversified paths driving the high-quality development of enterprises into four configuration paths:

#### 4.2.1 Technology innovation outstanding type.

The pathway characterized by prominent technological innovation is more applicable to enterprises that possess a high capability for R&D investment, are committed to driving technological innovation through digital technologies, and pursue high innovation output, so as to gain a competitive edge in the market and achieve high-quality development. This type corresponds to path P4 in period 3 of Table 6. Both configuration 4a and configuration 4b take high R&D investment, high innovation output, and no high government innovation subsidies as their core conditions. Specifically, this path mainly consists of three parts: digital technology, R&D investment, and innovation output. It emphasizes the investment and output of enterprises in technological innovation capabilities, so as to stand out in the highly competitive market, provide higher-quality products and services, and promote the high-quality development of enterprises. Thus, the article defines the coordinated development model of “digital technology”, “R&D investment” and “innovation output” as the “path with prominent technological innovation”. This path emphasizes the application and investment of enterprises in the field of digital technology, promoting technological innovation through high R&D investment, ultimately achieving high innovation output, forming a virtuous cycle, and facilitating high-quality development of enterprises.

Under the configuration path that highlights technological innovation, taking Wanma Co., Ltd. as an example, the company was founded in 1989. After more than 30 years of development, it has grown into a key national high-tech development enterprise. Wanma Co., Ltd. has profound technological accumulation and strong innovation capabilities in cutting-edge fields such as new manufacturing, new materials, and new energy, and is in a leading position in the industry. The company has always regarded technological research and development as its primary goal, adhering to the concept of “Innovation leads the future”, constantly improving products and processes, enhancing product performance and quality, to meet the constantly changing market and customer demands. The R&D team of Wanma Co., Ltd. is composed of a group of high-quality professionals. They closely follow the development trends of the industry, constantly explore new technologies and new processes, and promote product innovation and industrial upgrading. The company has established a complete R&D system and innovation mechanism, encouraging employees to carry out technological innovation and the transformation of achievements, thus creating a favorable atmosphere for innovation. Through continuous technological innovation, Wanma Co., Ltd. has developed a series of core technologies and products with independent intellectual property rights in the field of new intelligent manufacturing, such as intelligent production lines and automated equipment, providing strong support for the intelligent upgrade of the manufacturing industry. Apart from Wanma Co., LTD., the other three enterprises – TCL Zhonghuan, Zhongtong Bus, and Sinoma International – have also achieved remarkable success under the configuration path that highlights technological innovation. TCL Zhonghuan continuously innovates in the fields of semiconductor materials and photovoltaic new energy. Through high investment in research and development and technological innovation, it constantly enhances product performance and market competitiveness. Zhongtong Bus has been making continuous breakthroughs in the field of new energy buses, developing a series of new energy bus products with independent intellectual property rights, and promoting the development of the new energy vehicle industry. In the fields of new materials and engineering technology services, Sinoma International has continuously improved the quality of its products and services through technological innovation and R&D investment, promoting the high-quality development of related industries.

#### 4.2.2 “Technology + policy” two-wheel drive type.

The “technology + policy” dual-driven pathway is more suitable for enterprises that, with strong government policy support, leverage policy incentives to alleviate financial burdens, acquire R&D resources, and subsequently enhance their own technological innovation capabilities. This enables them to maintain a competitive edge, explore new markets, and promote high-quality development of the enterprises. This type corresponds to path P5 in period 3 of Table 6, which indicates that with the support of government policies, enterprises can achieve significant innovation results and competitive advantages by striving to enhance their technological innovation capabilities, even in the face of the challenge of insufficient talents. This path emphasizes the significant impact of the policy environment on enterprises’ innovation activities, as well as the possibility for enterprises to enhance their innovation capabilities through their own efforts with policy support. Specifically, with the support of government policies, enterprises can obtain various forms of support such as tax preferences and government innovation subsidies. These policy supports can not only alleviate the financial burden of enterprises, but also provide them with more resources and opportunities for technological innovation and R&D investment. For instance, tax incentives can reduce the research and development costs of enterprises, enabling them to have more funds for technological research and development and innovation activities. Government innovation subsidies can directly provide research and development funds for enterprises, supporting their investment in key technology research and development and innovation projects. Under this path, through the synergy of policy support and their own efforts, enterprises can not only enhance their innovation capabilities and maintain competitive advantages, but also explore new markets, enter new fields, and meet the constantly changing needs of customers.

Under the dual-wheel driven configuration path of “technology + policy”, taking Jiesai Technology as an example, since its establishment, the company has been committed to technological innovation and high-quality development. By constantly improving the production process, enhancing the capacity for independent innovation in scientific research and production, upgrading production equipment and raising the level of technological innovation, Jiesai Technology has achieved rapid development in its technological level and innovation capabilities. The company has not only achieved remarkable results in technological research and development and product innovation, but also performed outstandingly in market expansion and customer satisfaction, earning wide market recognition and customer trust. Meanwhile, Jiesai Technology actively responded to the policy call and fully utilized policy support such as tax incentives and government innovation subsidies. Jiesai Technology has reduced its R&D costs by enjoying tax preferential policies, enabling the company to have more funds available for technological research and development and innovation activities. Jiesai Technology has also obtained direct research and development financial support by applying for government innovation subsidies. Government innovation subsidies not only provide enterprises with research and development funds, but also offer them technical support and resource guarantees.

#### 4.2.3 “Technology + talent” dual-wheel driven type.

The “technology + talent” dual-driven pathway is particularly well-suited for enterprises operating in an environment lacking significant external policy support. These enterprises are capable of establishing talent attraction mechanisms and innovative management systems on their own to gather high-skilled talents. Relying on the advantages of these talents and their technological accumulations, they rapidly respond to market demands, establish competitive barriers, and drive the independent innovation and development of the enterprises. This type corresponds to paths P2 and P6 in period 3 in Table 6, which indicates that even without the support of government policies, enterprises can still achieve significant innovation results and competitive advantages by striving to enhance their technological innovation capabilities and recruiting a sufficient number of high-tech talents. This path highlights the decisive role of the endogenous driving force of enterprises in innovation activities, as well as the possibility of breaking through resource constraints through the aggregation of technical talents. Specifically, in the absence of external policy support, enterprises can form a unique innovation ecosystem by establishing a talent attraction mechanism, improving the innovation management system and deepening the collaborative cooperation among industry, academia and research. Under this path, enterprises rely on their talent advantages and technological accumulation to form a self-reinforcing innovation closed loop: technological breakthroughs attract more high-end talents to join, and the aggregation of talents further accelerates technological iteration. By building data-driven R&D decision-making systems, enterprises can accurately identify market demands and technological trends, and develop disruptive products or services. Such innovations can not only respond quickly to market changes, but also establish industry standards through the first-mover advantage in technology and form competitive barriers. Therefore, the article refers to the independent innovation model driven by marketization and relying on talents and technologies as the “technology + talent” dual-wheel driven path.

Under the dual-wheel drive configuration path of “technology + talent’, taking Zhongtai Chemical as an example, its vision is to become a chemical cotton spinning industry group led by innovation and with high-quality development. Zhongtai Chemical focuses on the fields of chlor-alkali chemical engineering and new materials. By building a collaborative system of “independent innovation + high-end talents”, it promotes the deep integration of technological research and development and industrial upgrading. The company has established a full-chain innovation platform covering “basic research - application development - technology transfer”, relying on the national-level enterprise technology center and postdoctoral workstation, attracting top scientific research talents at home and abroad, and forming a research and development team centered on polymer materials and green processes. To strengthen talent support, Zhongtai Chemical has implemented the “Double Hundred Talent Project”. Through equity incentives, project dividends and the “Challenge and Response” mechanism, it has introduced 43 high-level technical experts within three years, among whom 6 have been selected for national talent programs. In terms of the transformation of technological achievements, Zhongtai Chemical has established an agile innovation system of “market demand - technological breakthroughs - industrial incubation”. In response to the upgrading demands of the cotton spinning industry chain, the company has integrated the viscose fiber R&D team to develop differentiated fiber products with antibacterial and flame-retardant functions, increasing the added value by 30% and successfully entering the high-end medical textile market.

#### 4.2.4 Innovative comprehensive body type.

The comprehensive innovation – integrated pathway is more applicable to strategic emerging enterprises that can fully integrate resources from three aspects: government policy guidance, support from high – quality talents, and technological innovation investment. By leveraging the synergistic interaction among these three elements, these enterprises create a favorable development ecosystem. This enables them to reduce operational costs, enhance competitiveness, and achieve sustainable high – quality development. This type corresponds to paths P1, P3 and P7 in period 3 of Table 6. This reflects the coexistence of government, talent and innovation, and their coordinated matching, which can jointly promote the improvement of high-quality development of strategic emerging enterprises. By creating a positive policy environment, the government can provide enterprises with a stable business environment and market opportunities to reduce their operating costs and risks. Through policies and plans to introduce and cultivate high-quality talents, the government can help enterprises meet their talent demands. By supporting R&D investment, establishing innovation centers and laboratories, etc., the government can help enterprises enhance their technological level and innovation ability. The combined effect of the three can help enterprises reduce production costs, expand markets, enhance competitiveness, provide support and opportunities for the sustainable development of enterprises, and promote the high-quality development and improvement of enterprises. Therefore, the article names the innovation path that integrates “technology + talent + policy” as the “Innovation Comprehensive type” path.

Under the configuration path of the innovative comprehensive type, taking Pinggao Electric as an example, in terms of technological innovation, it has always adhered to the strategic guideline of “market-oriented, enhancing independent development capabilities and introducing innovative technologies simultaneously, and integrating industry, academia and research”, strictly promoting product renewal and upgrading as planned, and strengthening technological, management and business model innovation. In terms of talent innovation, implement major product research and development awards to motivate the enthusiasm of R&D personnel. Actively implement the talent construction project to enable on-the-job R&D personnel to learn new technologies. Meanwhile, through the independent development of a series of products, cultivate a group of R&D personnel with strong business capabilities, high professional qualities and rich experience. The company enhances the technical and innovation capabilities of its employees through talent introduction and cultivation, making up for the shortage of talents and improving the technological innovation capabilities and market competitiveness of the enterprise. In terms of policy innovation, we will vigorously promote strategies such as technological innovation and new energy, implement the “dual carbon” goals, and comprehensively promote the further transformation of XJ Electric into a modern and intelligent enterprise. Through policy support and government guidance, the company acquires more resources and opportunities for technological innovation and R&D investment, promoting the high-quality development of the enterprise. For instance, with the support of policies, the company has established multiple innovation centers and laboratories, carried out a number of key technology research and development and innovation projects, and achieved remarkable technological breakthroughs and innovation results. Under the impetus of policies, the technical and talent levels work together and promote each other, helping enterprises maintain a professional level of technology, have sufficient R&D personnel to expand business, and at the same time ensure a continuous flow of funds for enterprises, thereby helping enterprises achieve high-quality development.

A further horizontal analysis of the results reveals that, first of all, at the technical level, the technical level of enterprises in all three paths is a core variable, and the R&D investment and innovation output at the technical level have a certain substitution effect. The comparison of Path 1, Path 2 and Path 3 shows that to achieve high-quality development of an enterprise, at least one of the two needs to be met. Specifically, Configuration 1 indicates that as long as an enterprise simultaneously possesses high R&D investment and high innovation output conditions, it can achieve high-quality development. This implies that forming a significant technological progress advantage can, to a certain extent, make up for the deficiencies at the talent and policy levels. Secondly, the R&D investment at the technical level in the four configurations is a core condition for the high-quality development of strategic emerging enterprises. This further proves that for enterprises to achieve high-quality development, they must attach importance to technological innovation and R&D investment. Whether in a technology-driven, talent-driven, innovation-integrated or other configuration paths, enterprises must continuously enhance their technological level and innovation capabilities through high R&D investment and high innovation output, develop new products and technologies with market competitiveness, and meet the constantly changing market and customer demands. Finally, in terms of talent innovation and policy innovation, except for configuration 1, the proportion of highly educated talents and tax incentives in the other three configurations are all core conditions for strategic emerging enterprises to enhance high-quality development. This indicates that for enterprises to achieve high-quality development, they must attach great importance to the introduction and cultivation of talents, enhance the technical and innovation capabilities of their employees. Meanwhile, for the government to support strategic emerging enterprises in achieving high-quality development, it must increase tax incentives, reduce the financial burden on enterprises, and provide them with more resources and opportunities for technological innovation and R&D investment.

### 4.3 Multi-period comparative analysis

First of all, observe the changes of individual antecedent conditions in each period. On the one hand, R&D investment plays a very important role within the research sample range. In the configuration of the three time periods, R&D investment is a core condition, indicating the stability and importance of R&D investment in the high-quality development of strategic emerging enterprises. On the other hand, the importance of innovation output began to emerge in period 3 and appeared as a core condition six times. It appears as an auxiliary condition once.

Secondly, observe the interrelationships among the antecedent conditions in each period. The synergy model of R&D input and innovation output shows significant stability during the evolution process, and the two form a two-way reinforcement mechanism. Research and development investment provides material guarantee for the continuous emergence of innovative outputs by building the resource basis of an enterprise’s innovation ability. The accumulation of innovative outputs feeds back to the improvement of R&D efficiency, forming a spiral upward cycle of “input - output – reinput”. Research and development investment is one of the important driving forces for innovation output. The two are closely related, mutually reinforcing and jointly promoting the development of new quality productive forces.

Finally, observe the number of configuration paths in each period. By comparing the configuration paths of Period 1, Period 2 and Period 3, it can be found that the configuration for achieving high-quality development first increased from one path in period 1 to two paths in period 1, and then to four paths in period 3. This not only reflects the increase in practical paths for achieving high-quality development of strategic emerging enterprises, but is also closely related to China’s active implementation of policies to promote their high-quality development.

### 4.4 Robustness test

The article conducts the robustness test by increasing the PRI consistency threshold [46,47], that is, reducing the PRI threshold value from 0.8 to 0.75. The results in Table 7 show that the overall consistency and coverage have only undergone minor changes, there are no obvious fluctuations in the overall results, and there is an obvious subset relationship among each configuration [29], indicating that the research conclusion of this article has high robustness.

**Table 7:** Robustness test for adjusting the PRI consistency threshold.

Variables	Time1		Time2						Time3					
	P1	P2	P3		P4	P5			P6			P7	P8	
	H1	H2	H3a	H3b	H4	H5a	H5a	H5c	H6a	H6b	H6c	H7	H8a	H8b
DT	⨂	⨂		⨂		●	●		⨂	⨂	⨂	H7	H8a	H8b
RDI	●	●	●	●	●	●	●	●	●	●	●		●	
IO	●	●	●	●	●	●	●	●	●	●	●	●	●	●
RDP		●	●		⨂		●			⨂	⨂	●	●	●
PHEP	●		●	●		●		●	⨂		⨂			●
TB		⨂			●		⨂	●					●	●
GIS	●	●		⨂	⨂	●	●		⨂	⨂		●	⨂	●
CS	0.94	0.93	0.94	0.93	0.94	0.94	0.93	0.96	0.96	0.95	0.96	0.96	0.95	0.96
RC	0.49	0.51	0.43	0.46	0.43	0.36	0.33	0.26	0.34	0.31	0.39	0.30	0.32	0.30
UC	0.06	0.08	0.02	0.01	0.02	0.03	0.00	0.01	0.02	0.01	0.05	0.00	0.06	0.01
OU	0.92		0.92						0.94					
OC	0.57		0.62						0.60					

**Note:** ● indicates the presence of a core condition, ● indicates the presence of an edge condition, ⨂ indicates the absence of a core condition, ⨂ indicates the absence of an edge condition, and a blank indicates the presence or absence of a condition.

### 4.5 Further analysis

This paper first uses the traditional empirical regression causal model $Y=a+b_{1}X_{1}+b_{2}X_{2}+b_{3}X_{3}+\cdots$ to verify the correlation between variables and high-quality development. In this paper, K-means cluster analysis is carried out on 7 characteristic variables, and the number of clusters is determined by combining elbow method and the distribution characteristics of sample data. When the number of clusters k is set to 1 or 2, the decrease of the sum of squares in clusters is very significant. This indicates that with the increase of clusters, the degree of aggregation of data points is rapidly improved, and the similarity between data points in each cluster is significantly enhanced. However, once k exceeds 3, the variation amplitude of squares and CSS in clusters gradually decreases, indicating that increasing the number of clusters does not significantly improve the partitioning of data points, that is, the optimal cluster number may be close to 2. When k = 2, the value of the Silhouette Coefficient is optimal, further verifying the ideality of the clustering effect. The profile coefficient is an important index to measure the clustering quality. A higher value indicates better clustering effect, stronger cohesion of data points in their own clusters, and greater differentiation from data points in other clusters. Therefore, this paper decides to set the number of clusters to 2. Finally, by learning the sample data of 176 feature variables, we successfully identify 2 groups with similar features (i.e., “clusters”).

**Fig 3:**
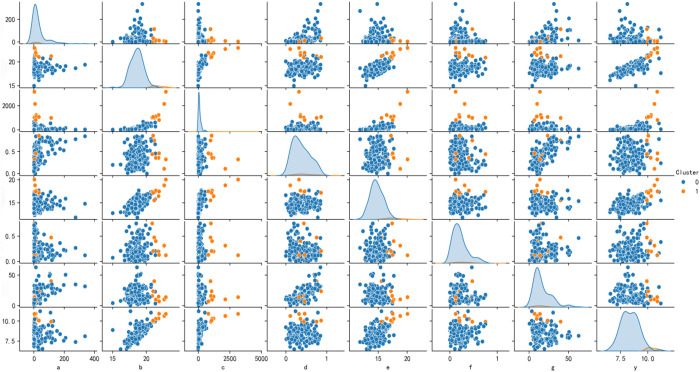
Scatter matrix of the correlation between each antecedent variable and high-quality development.

Fig 3 shows the correlation between the two classes of antecedents and the scatter matrix of the relationship between a single antecedent variable and high-quality development. First, there is an overall trend of positive correlation between the antecedents, which indicates that when a certain antecedent variable increases, other antecedents may also rise with it, reflecting possible synergies between them. However, the relationship between some variables is not very clear, which lays a foundation for further exploration of the influence of different combinations of antecedents on high-quality development. Then, through a systematic analysis of multiple variables, we find that there is a certain degree of positive correlation between the seven characteristic variables examined and high-quality development. This finding suggests that the factors that promote high-quality development are not determined by a single antecedent variable, and many other factors also play an important role. Finally, from the perspective of classification, the relationship between different antecedent variables and high-quality development becomes clearer. This shows that through cluster analysis, we can effectively separate research objects with similar characteristics from complex non-linear environments. This process not only simplifies the research question, but also enables subsequent research to focus more on the specific effects of different antecedents on high-quality development, thus supporting the formulation of more precise policies and strategies.

Traditional empirical regression models usually assume that the absence of one antecedent variable can be compensated for by increasing the value of other antecedents. However, this view actually fails to fully account for the combined effects between the various conditions and their impact on the outcome. In many cases, the interaction of conditions can affect the presentation of the final outcome, and the addition of a single antecedent variable is not an effective substitute for the missing factor. Therefore, relying solely on traditional regression models can lead to a one-sided understanding of causality. Therefore, a more representative model, $Y=f\left(X_{1}\times X_{2}\times X_{3},\cdots \right)$, is chosen in this paper. In this way, the essay is able to articulate more clearly the interdependence of the individual antecedents and how they work together to affect the final result. Such an analysis not only complements the traditional regression model, but also provides a more comprehensive and in-depth understanding of the framework for studying causality in complex systems.

The data were imported into stata18 software for descriptive statistical analysis, correlation analysis and multicollinearity test of variables (as shown in Table 8). It can be seen from the table that 1/VIF are all less than 1, that is, there is no multicollinearity among the antecedents, and there is a significant positive correlation between R&D input, innovation output, and the proportion of highly educated personnel and high-quality development. It can be assumed that the three variables are the core influencing factors of the research results.

**Table 8:** Descriptive statistics, correlation analysis and multicollinearity test.

Variables	(1)	(2)	(3)	(4)	(5)	(6)	(7)	(8)
DT	1							
RDI	−0.0410	1						
IO	0.751***	0.0670	1					
RDP	0.555***	−0.0780	0.687***	1				
PHEP	0.0590	0.577***	0.210***	0.0170	1			
TB	0.537***	−0.0440	0.710***	0.615***	0.0960	1		
GIS	0.0930	−0.0410	0.151**	0.127*	−0.165**	0.112	1	
DT	−0.100	0.577***	0.0720	−0.0370	0.713***	0.00700	−0.00100	1.000
Mean	29.55	18.74	170.2	0.379	14.84	0.222	16.42	8.461
Standard deviation	50.89	1.16	341.2	0.213	1.281	0.167	10.99	0.914
VIF	–	1.67	2.79	2.09	2.56	2.17	1.11	2.32
1/VIF	–	0.600	0.358	0.479	0.391	0.462	0.900	0.431

**Note:** ***p < 0.01, **p < 0.05, *p < 0.1.

Since there is a significant positive correlation between “R&D investment” and “innovation output”, in order to avoid potential interference with subsequent conclusions, the article makes “R&D investment” and “innovation output” alternate, and conducts multiple linear regression tests on the data in different cases. The results are shown in Table 9. When both “R&D investment” and “innovation output” exist simultaneously, it can be found that “R&D investment” shows the most significant positive correlation with the results. However, when retaining “innovation output” and eliminating “R&D investment”, multiple anedent variables including “innovation output” showed a significant positive correlation with the results (all P values were less than 0.10); Even when “R&D investment” is retained and “innovation output” is excluded, it is still “R&D investment” that shows the most significant positive correlation with the results. The above research conclusions are consistent with the results obtained by fsQCA, that is, R&D investment is the core condition for strategic emerging enterprises to achieve high-quality development.

**Table 9:** Results of multiple linear regression.

High-quality development			
	(1)	(2)	(3)
Digital technology	−0.000	0.001	0
	(−0.07)	−0.46	(−0.13)
R&d investment	0.580***	–	0.604***
	(8.92)	–	−10.54
Innovation output	0.000	0.001***	–
	(0.78)	−4.67	–
R & D personnel	0.082	0.773*	0.055
	(0.24)	−1.93	−0.16
Proportion of highly educated people	−0.013	0.214***	−0.003
	(−0.25)	−3.91	(−0.07)
Tax benefits	−0.112	0.247	−0.113
	(−0.40)	−0.72	(−0.40)
Government innovation subsidies	−0.013**	−0.020***	−0.013**
	(−2.14)	(−2.62)	(−2.12)
_cons	−2.032*	5.082***	−2.586***
	(−1.97)	−6.46	(−3.47)
N	176	176	176
R^2^	0.59	0.395	0.317
adg.R^2^	0.572	0.374	0.297

**Note:** ***p < 0.01, **p < 0.05, *p < 0.10, standard error in parentheses.

## 5 Conclusion and enlightenment

### 5.1 Research conclusion

This article takes 176 strategic emerging enterprises listed on the A-share market as research cases. It employs a combination of NCA with multi-period QCA and empirical regression analysis to summarize practical pathways for enhancing the high – quality development of strategic emerging enterprises, leading to the following conclusions:

The results from the NCA and multi-period QCA analyses indicate that the seven independent variables lack sufficient explanatory power when considered individually. However, by conducting a horizontal comparison of the driving mechanisms across the three periods, it becomes evident that improving technological capabilities plays an indispensable role in achieving high-quality development for strategic emerging enterprises. Enterprises should prioritize technological innovation, allocate substantial resources to R&D funding, and persistently engage in deep exploration of specialized fields. Leading technological advancements within the industry is crucial for strategic emerging enterprises to enhance their overall development quality.

Multiple linear regression reaffirms that “R&D investment” is the core and critical factor influencing the high-quality development of strategic emerging enterprises. Additionally, “innovation output,” “the proportion of highly educated talents,” and “tax incentives” also play a supportive role in promoting high-quality development. The fsQCA results further confirm that “R&D investment” serves as the central condition driving the high-quality development of strategic emerging enterprises. Together, these two analytical methods corroborate that R&D investment is the most pivotal element for achieving high-quality development in this sector.

Configuration analysis elucidates the mechanisms by which technological innovation, talent innovation, and policy innovation jointly propel the high-quality development of strategic emerging enterprises at three distinct levels. In period 1, there exists one configuration characterized by comprehensive innovation. In period 2, two configurations emerge: the “technology + policy” dual-driven type and the innovative comprehensive type. By period 3, four configurations are identified: the type dominated by technological innovation, the type driven by both “technology and talent,” the type driven by both “technology and policy,” and the comprehensive innovation type. Throughout all periods, R&D input and innovation output consistently play crucial roles, while other condition variables exhibit varying degrees of influence within each configuration.

### 5.2 Research implications

The interplay among various factors reflects the intricacy of high-quality development in strategic emerging enterprises. Enterprises should leverage their own developmental advantages and resource endowments to foster high-quality growth through the synergistic effects of multiple factors along an effective trajectory. Building upon the research findings, this paper puts forth the following enlightenments:

(1) Technology plays a pivotal role in facilitating high-quality development. Based on research findings, it is evident that technological innovation serves as an indispensable and powerful driver for strategic emerging enterprises to achieve high-quality development at all times. Without reaching a certain level of technological innovation, enterprises may encounter a “bottleneck” effect, making it challenging to achieve high-quality development even with sufficient high-quality talent and government support. Currently, strategic emerging enterprises face significant technical challenges. For example, the average investment intensity in basic research among China’s strategic emerging enterprises is only 1.8%, compared to 7.6% for Intel Corporation in the United States. Additionally, China’s industrial chain exhibits excessive dependence on key technologies, with many critical materials requiring imports. Therefore, enterprises should prioritize core technologies, pursue revolutionary breakthroughs through disruptive and cutting-edge innovations, enhance their strategic scientific capabilities, establish systems for the transformation of scientific and technological achievements, promote the effective conversion of these achievements, and ultimately contribute to high-quality development.
(2) Government-enterprise collaboration to build a high-quality platform for innovative talents. The government and enterprises should embrace the concept that innovative talents are the primary resource, formulating open talent introduction policies tailored to the specific needs of strategic emerging enterprises. Drawing inspiration from Singapore’s Tech@SG initiative, the government could provide subsidies and incentives to attract top-tier talents in cutting-edge fields such as artificial intelligence and quantum computing, thereby enhancing its capacity to recruit and retain high-caliber professionals. Simultaneously, enterprises should refine their employment mechanisms for innovative talents, intensify talent development efforts, prioritize an income distribution system oriented toward innovation, implement a competitive salary and reward structure, and stimulate the innovation potential and enthusiasm of employees. Furthermore, the government and enterprises should collaborate to establish practical platforms for cultivating high-quality skilled talents, offering robust support for the growth of strategic emerging enterprises and the advancement of future industries. The government can contribute by providing venues and funding, while enterprises can offer hands-on opportunities and training resources. Both parties can jointly establish talent training bases and innovation laboratories, creating a conducive environment for talent development and fostering practical experience. For example, the government can partner with enterprises to create high-level innovation and entrepreneurship hubs and incubators, delivering services such as business mentoring and financial assistance to talents, thereby promoting their innovation and entrepreneurship capabilities and driving high-quality development.
(3) Change thinking and fully leverage the collaborative role of multiple stakeholders. To strengthen the two-way interaction between the government and enterprises, the government may introduce a series of tax incentive policies, such as additional deductions for R&D expenses and preferential tax rates for high-tech enterprises, to reduce the innovation costs and risks faced by enterprises and incentivize them to increase investment in technological innovation. The government should also provide innovation subsidies and special funding support to assist enterprises in overcoming financial constraints and promoting technological advancement and industrial upgrading. Meanwhile, enterprises need to closely monitor policy changes, actively align with national strategies, secure government financial support, and increase investment in technological innovation. Based on their own development advantages and resource endowments, they should select appropriate and practical innovation pathways to drive high-quality development. School-enterprise collaboration should transcend the traditional internship base model. For instance, it could adopt the “School-Enterprise Joint Laboratory” model implemented in Shenzhen, where enterprises propose specific R&D demands and contribute 50% of the funding, while university teams conduct on-site research and development to establish an intellectual property sharing mechanism. The government can formulate supportive policies to encourage universities and enterprises to engage in industry-education integration, jointly develop teaching resource libraries, and provide high-quality educational resources and practical opportunities for talent cultivation.

### 5.3 Limitations and prospects of the study

This paper has the following limitations: First, regarding the research sample size, the study is confined to strategic emerging enterprises, which may not fully capture the driving mechanisms of high-quality development for enterprises in other industries. Future studies could extend the scope to include more industry-specific enterprises, thereby enhancing the generalizability of the findings. Second, concerning the choice of research methods, this study employed a combination of NCA, multi-period fsQCA, and empirical regression analysis to reveal the combined effects of driving factors. While temporal characteristics were incorporated, alleviating errors associated with sample time selection to some extent, it remains challenging to precisely assess differences between configurations at each time point and the overall configuration using inter-group consistency distance and intra-group consistency distance as in panel QCA. More in-depth discussions on this aspect are warranted in future research. Finally, during the regression analysis, no moderating variables were included. Future studies could explore the moderating effects of additional factors, offering more targeted recommendations for promoting the high-quality development of strategic emerging enterprises.
